# Are We Overusing Coagulation Studies in the Emergency Department?

**DOI:** 10.1155/2024/8694183

**Published:** 2024-04-23

**Authors:** Bader Alyahya, Abdulaziz Alalshaikh, Ali Alkhulaif, Tareq Al-Salamah, Badr Aldawood, Alwaleed Alsubaie, Meshal Alohali, Saud Alshenaifi, Abdulaziz Alohali, Majed B. Alzin, Abdullah Almana, Mohammed Habib, Rana Hasanato

**Affiliations:** ^1^Department of Emergency Medicine, College of Medicine, King Saud University, Riyadh, Saudi Arabia; ^2^King Abdullah University Hospital, Riyadh, Saudi Arabia; ^3^Department of Emergency Medicine, Prince Sultan Military Medical City, Riyadh, Saudi Arabia; ^4^Medical Services Saudi Royal Guard, Riyadh, Saudi Arabia; ^5^College of Medicine, King Saud University, Riyadh, Saudi Arabia; ^6^Laboratory Medicine Department, College of Medicine, King Saud University, Riyadh, Saudi Arabia

## Abstract

**Methods:**

This retrospective observational study, conducted in the ED of King Saud University Medical City (KSUMC) in Riyadh, Saudi Arabia, during July and August of 2021(2 months) examined coagulation profile requests. Patients' demographic data (age and gender), medical and clinical history (presenting complaint, comorbidities, and diagnosis), the use of antiplatelets or anticoagulant agents and laboratory values for PT, APTT, and INR were collected. We calculated the total cost of unnecessary coagulation profile testing based on the independent assessment of two ED consultants.

**Results:**

Of 1,754 patients included in the study, 811 (46.2%) were males and 943 (53.8%) were females, with a mean age of 42.1 ± 18.5 years. There were 29 (1.7%) patients with liver disease and 21 (1.2%) patients had thromboembolic disease. The majority of the patients' results were within normal levels of PT (*n* = 1,409, 80.3%), APTT (*n* = 1,262, 71.9%), and INR (*n* = 1,711, 97.4%). Evidence of active bleeding was detected in 29 patients (1.7%). Among patients with bleeding only one had an abnormal INR (3.01) and was on warfarin. Forty-six (2.6%) patients had elevated INR level. Cohen's kappa between the two consultants was recorded at 0.681 (substantial agreement) in their assessment of the appropriateness of coagulation tests requests and both consultants believed that 1,051 tests (59.9%) were not indicated and were unnecessary. The expected annual cost saving if the unnecessary tests were removed would be around SAR 1,897,200 (approximately US$ 503,232) which is about SAR 180000 (US$ 48000)/1000 patients.

**Conclusion:**

This study showed that coagulation tests are overused in the ED. More than half of coagulation profile tests in our study population were deemed unnecessary and associated with significant cost. Targeted testing based on specific patient presentation and medical history can guide physicians in wisely choosing who needs coagulation studies.

## 1. Introduction

Blood tests are often requested indiscriminately in the Emergency department (ED) and many EDs have preset orders that are usually performed during triage before performing clinical assessment to improve the flow of patients through the department [1]. Conducting such tests might expedite patient care and reduce waiting time in the ED. However, it should be noted that if unnecessary, this process may overburden the laboratory department, delay the disposition of the patient, and add avoidable cost [2, 3]. Besides, it is expected that some of the randomly conducted blood tests will not contribute to the diagnosis and management of the condition [4]. Identifying these unnecessary tests in the emergency department can significantly save resources without adversely impacting patients' outcomes.

Coagulation profile testing consists of prothrombin time (PT), partial thromboplastin time (PTT), and international normalized ratio (INR) [5]. Evidence shows that coagulation profile testing is useful in certain circumstances including patients with active bleeding or high risk of bleeding, presence of petechia or ecchymoses [6]. PT/INR is indicated for patients with liver disease or severe systemic illness including sepsis, disseminated intravascular coagulation, preeclampsia, or patient on warfarin therapy and before administration of thrombolytics. However, in 2014, it was recommended against using coagulation testing in the emergency department for cases without bleeding or suspected coagulopathy [7, 8]. Monitoring coagulation profile is useful in adjusting anticoagulant dosing; however, it is not usually necessary before the initiation of anticoagulation medication in the ED [9]. Several studies have suggested that routine coagulation profile assessment does not impact the clinical management in the ED [3, 7, 10–12].

We conducted this study to evaluate the frequency of coagulation profile requests and the incidence of abnormal coagulation profiles in our institution, and evaluate what proportion of these are unnecessary for patient care.

## 2. Methods

This was a retrospective observational study conducted in the ED of King Saud University Medical City (KSUMC) in Riyadh, Saudi Arabia. We enrolled all adult and pediatric patients admitted to the ED between July and August of 2021 (2 month period) who had coagulation profile requests. We excluded patients who did not have coagulation profile testing results. Patients' demographic data (age and gender), medical history (presenting complaint, past medical history/comorbidities, and diagnosis with special focus on presentation and risk factors that may suggest bleeding or coagulopathy), their use of antiplatelets or anticoagulants, and the laboratory values for PT, APTT, and INR were collected in a predetermined data collection sheet. Direct acting oral anticoagulants (DOACs) levels are not available in our institution and therefore were not assessed. Based on the clinical presentation and medical history, two Emergency Medicine board-certified consultants (BA) and (AA) with more than 5 years of experience as attending ED physicians, independently reviewed the cases to determine based on the previously mentioned indicators whether the coagulation profile testing was indicated or not. If any of the consultants thought the test was indicated, it was considered necessary (please refer to Supplementary Material 1 for the detailed description of the process of assessment of the appropriateness of coagulation tests requests). We calculated the total costs of coagulation profile (PT, aPTT, and INR) tests deemed unnecessary, by multiplying the number of tests by the average cost of a coagulation profile from 4 private and governmental hospitals in Saudi Arabia (approximately 300 SAR (US $80)).

Data was analyzed using the Statistical Package for Social Sciences (SPSS) version 26.0 (IBM-SPSS Inc., Armonk, New York, USA). Results are presented as numbers and percentages for categorical variables and as mean and standard deviation for continuous variables. A test of agreement (inter-observer agreement) was done using the Cohen's kappa statistics [13]. The Institutional Review Board (IRB) of the Deanship of Scientific Research, College of Medicine, King Saud University in Riyadh, Saudi Arabia, granted the ethical approval to conduct the study (Project No. E-21-6196). Informed consent waiver was obtained given the retrospective nature of the study and all patient data were kept strictly confidential.

## 3. Results

A total of 3,218 patients had blood work done during our study period, we included 1,754 (55%) patients who had coagulation profile requested, 811 (46.2%) males and 943 (53.8%) females, with a mean age of 42.1 ± 18.5 years (range: 1 to 98 years old). The most prevalent presenting symptoms were gastrointestinal- (GI-) related (most commonly abdominal pain) in 392 (22.3%) patients followed by CNS-related complaints in 169 (9.6%) patients. No associated comorbid condition was noted in 696 (39.7%) patients. However, among patients with comorbid conditions, diabetes mellitus was the most prevalent (*n* = 417, 23.8%), followed by hypertension (*n* = 386, 22.0%). 29 patients (1.7%) had liver disease and 21 patients (1.2%) had thromboembolic disease. 173 patients (9.8%) were on aspirin and 96 patients (5.5%) on anticoagulants.  Table 1 shows the detailed clinical and demographic profiles of all patients.

**Figure 1:**
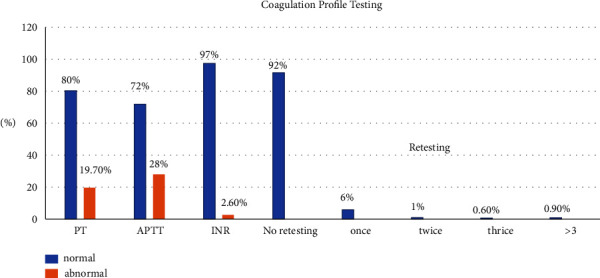
Coagulation profile testing and retesting results.

The majority of coagulation profile results were within normal levels of PT (*n* = 1,409, 80.3%), APTT (*n* = 1,262, 71.9%) and INR (*n* = 1,711, 97.4%). Retesting for INR was done in 148 (8.4%) patients (Figure 1). Evidence of active bleeding was present in 29 patients (1.7%), the most frequent cause being gynecological (58.6%), followed by hematological (10.3%), then GI and nasal bleeding (6.9%). However, only one patient of those with bleeding had an abnormal INR (3.01), he was on warfarin because of history of mitral valve repair surgery. Tranexamic acid was administered to control the bleeding in 11 patients (37.9%). Factor 9 was used in one patient with hemophilia A whose INR was 1.05, PT and APTT were 14.6 and 66.2, respectively.

Forty-six patients (2.6%) had elevated INR levels. Factors associated with elevated INR are: the use of warfarin (OR = 163.4, 95% CI = 66–404.8, *p* < 0.001), direct oral anticoagulants (OR = 6.80, 95% CI = 3.24–14.3, *p* < 0.001), liver disease (OR = 4.513, 95% CI = 1.316–15.485, *p*=0.017), age (OR = 1.040, 95% CI = 1.024–1.056, *p* < 0.001), and malignancy (OR = 3.033, 95% CI = 1.049–9.733, *p*=0.041). However, gender (OR = 1.634, 95% CI = 0.884–3.021, *p*=0.117) and comorbid conditions such as dyslipidemia, CAD, stroke, ESRD, thromboembolic disease, and hypothyroidism were not found to be associated with elevated INR (Table 2).

INR test was repeated (in the ED or during admission) in 148 (8.4%) patients for a total of 277 repeated tests. Of these, 102 (36.8%) were deemed as not indicated. Consultant A noted 691 (39.4%) of the INR tests were indicated, whereas consultant B noted only 509 (29.0%) of the INR tests were indicated. Cohen's kappa between the two consultants was recorded at 0.681 (substantial agreement) in their observations of the INR results. Overall, both consultants believed that 1,051 tests (59.9%) were not indicated and were unnecessary, equating to approximately SAR 316,200 (approximately US$ 83,872) of unnecessary expenses. The expected annual cost saving would be around SAR 1,897,200 (approximately US$ 503,232) which is about SAR 180000 (US$ 48000)/1000 patients.

## 4. Discussion

Coagulation profile tests are requested when we suspect abnormal blood clotting. The sensitivity and specificity of coagulation tests specifically INR was reported at 94% and 88%, respectively [14]. However, the diagnostic performance of these tests differs when patients are on direct oral anticoagulants, in which the performance for diagnostic interpretation becomes limited [15].

In this study, we wanted to determine the incidence of abnormal coagulation profiles, the frequency of requests done, and the number of requests made that was deemed unnecessary. Our results showed that the incidence of abnormal INR in the ED was 2.6%, while abnormal PT was detected in 19.7% and abnormal PTT in 28%. This rate is lower than what has been reported in a similar study by Martin et al. [10], which demonstrated that 9.2% of their population had INR out of the reference range. On the other hand, another investigation by Schwartz et al. [16], showed that 13% had abnormal INR among patients with coronary artery syndrome. These studies demonstrated that the treatment regimens of patients with abnormal blood tests were not affected [10, 16]. On the other hand, a study showed that among patients on warfarin, and patients with liver failure, sepsis, and multiple organ failure, coagulation testing was associated with changing the clinical management in the ED [17]. These studies showed that coagulation testing is only useful when used in appropriately selected patients.

In this study, evidence of active bleeding was detected in only 1.7% of our patients, and only one of them had an abnormal INR who was on warfarin because of history of mitral valve repair surgery. This is significantly lower than what was reported in one study about warfarin related adverse events in the ED, major/minor bleeding were reported in 33.3% of patients [18], while in another study, the incidence of bleeding was 13.9% and among those with bleeding 50.0% had supratherapeutic INR [19]. Moreover, several studies have shown no change in the management plans of their patients despite the abnormal or normal coagulation profiles that were seen among their patients [9, 10]. The cost-effectiveness of coagulation profile testing in the ED has been investigated in many studies and several international committees do not encourage pre-procedure or pre-operative coagulation profile tests [20–24]. We suggest that a coagulation testing policy based on clinical presentation (active bleeding or bruising, stroke, or a condition necessitating anticoagulation), risk factors (known bleeding disorder, anticoagulants intake, and liver disease) is needed. Reflex and routine coagulation tests requests should be avoided.

On the contrary, warfarin use has been suggested as an indication for coagulation testing for patients presenting to the ED if clinically indicated [17]. Patients who have predictive factors for an abnormal coagulation profile may have coagulation profile testing including those with alcohol abuse, known liver disease, and anticoagulation therapy [16]. During the time of the CoVid-19 pandemic surge, many severe CoVid-19 patients had abnormal coagulation profiles, as severe CoVid-19 infection is more likely to develop coagulopathy than mild infections [24–26]. Furthermore, bleeding is not commonly encountered among these patients despite abnormal coagulation profiles [27–29]. Our study was conducted during COVID-19 pandemic peak, and we considered positive COVID-19 test as an appropriate indication of performing coagulation studies. However, many of these patients had mild COVID-19 symptoms and probably did not need the test. Hence, we believe that 59.9% is an underestimation of the number of unnecessary tests.

This study validates previous reports that suggested the need for increased attention to cost reduction and cost-effectiveness when requesting coagulation profile testing. Our findings approximated a potential cost reduction of US $ 83,872 over 2 months from avoiding unnecessary coagulation profile testing.

The retrospective nature of this study serves as its limitation since it may have introduced selection bias and temporal relationships may be difficult to assess. Also, data that may justify some of the unnecessary tests might be missing in the chart. Further, although physicians usually decide on doing certain investigations based on the clinical presentation at the time, a follow up assessment would have been helpful to ensure that none of the patients (whose tests were considered unnecessary) developed bleeding or thrombosis which might justify the test.

## 5. Conclusion

This study showed that we are overusing coagulation tests in the ED. More than half of coagulation profile tests in our study population were deemed unnecessary and associated with significant cost. Targeted testing based on specific patient presentation and medical history can guide physicians in wisely choosing who needs coagulation studies.

## Figures and Tables

**Table 1:** Clinical and demographic profile of 1,754 patients who were ordered coagulation profile testing.

Characteristics	*N* = 1,754
Age in years, mean ± SD	42.1 ± 18.5
Gender, *n* (%)	
Male	811 (46.2%)
Female	943 (53.8%)
Chief complaints	
GI related complaints	392 (22.3%)
CNS related/stroke	169 (9.6%)
Trauma	121 (6.9%)
Bleeding and bruising	117 (6.7%)
Dyspnea	96 (5.5%)
Palpitation	58 (3.3%)
Urinary symptoms	57 (3.2%)
Leg pain and swelling	54 (3.1%)
Fever	38 (2.2%)
Back pain	31 (1.8%)
Others	
Toxic ingestion	13 (0.7%)
Psychiatric symptoms	5 (0.3%)
None (sent to do coagulation profile)	9 (0.5%)
Comorbidities, *n* (%)	
None	696 (39.7%)
Diabetes mellitus	417 (23.8%)
Hypertension	386 (22.0%)
Dyslipidemia	167 (9.5%)
Coronary artery disease	96 (5.5%)
Malignancy	56 (3.2%)
End-stage renal disease	38 (2.2%)
Atrial fibrillation	29 (1.7%)
Liver disease	29 (1.7%)
Thromboembolic disease	21 (1.2%)
Ischemic stroke	20 (1.1%)
Hypothyroidism	76 (4.3%)
Others	
Systemic lupus erythematosus	8 (0.5%)
Cardiomyopathy	7 (0.4%)
Hemophilia A	3 (0.2%)
Antiphospholipid syndrome	3 (0.2%)
Carotid stenosis	1 (0.1%)
Medications used	
Aspirin	173 (9.8%)
Enoxaparin	37 (2.1%)
Rivaroxaban	29 (1.7%)
Warfarin	18 (1.0%)
Aspirin and clopidogrel	13 (0.7%)
Apixaban	10 (0.6%)
Others	
Heparin and warfarin	1 (0.06%)
Dabigatran	1 (0.06%)

**Table 2:** Factors associated with an elevated INR.

	OR	95% CI	*P* value
Use of warfarin	163.4	66.0–404.8	<0.001
Use of direct oral anticoagulants	6.80	3.24–14.3	<0.001
Presence of liver disease	4.513	1.316–15.485	0.017
Malignancy	3.033	1.049–9.773	0.041
ESRD	2.111	0.493–9.045	0.314
Dyslipidemia	2.051	0.940–4.473	0.071
Stroke	1.975	0.259–15.080	0.512
Coronary artery disease	1.673	0.587–4.766	0.335
Gender	1.634	0.884–3.021	0.117
Thromboembolic disease	1.289	0.878–1.893	0.196
Age	1.040	1.024–1.056	<0.001
Hypothyroidism	1.004	0.239–4.220	0.996

## Data Availability

The data that is used to support the findings of this study are available from the corresponding author upon reasonable request.

## Supplementary Materials

Supplementary Materials Supplementary Material 1: for detailed description of the process of assessment of the appropriateness of coagulation tests please refer to supplementary material 1.
