# Supplementary material for: Are We Overusing Coagulation Studies in the Emergency Department?
Source: Emerg Med Int. 2024 Apr 23;2024:8694183. doi: 10.1155/2024/8694183 (PMC11060872; doi:10.1155/2024/8694183)
Supplement: Supplementary Materials — Supplementary Material 1: for detailed description of the process of assessment of the appropriateness of coagulation tests please refer to supplementary material 1. [file 8694183.f1.docx]

**Supplementary Material 1:**

**Objective: to identify those who clearly do NOT need cogulation tests. If there is any doubt the cogulation test was considered necessary. This is probably an underestimation of the percentage of unncessary test.**

**Start:**

**1. Presenting Complaint Necessitates Coagulation Profile?**

- Examples: External/internal bleeding, hemodynamic instability, cardiac arrest, significant trauma, liver failure/cirrhosis, suspected or confirmed COVID-19, patient unable to provide history and no collateral history
- **Yes:** Coagulation Studies Necessary
- **No:** Go to Step 2

**2. Past Medical History Suggests Need for Coagulation Studies?**

- Bleeding disorder, coagulopathy, other relevant conditions (e.g DVT, PE, ECT)
- **Yes:** Coagulation Studies Necessary
- **No:** Go to Step 3

**3. Physical exam finding indicate the need for cogulation test: bleeding, brusing, etc.**

**Yes:** Cogulation tests necssary

**No:** Go to step 4

**4. Medication associated with Risk of Bleeding? ( again we know that many of those do not need coagulation studies but we rather underestimsate the percentage of unncessary tests than overestimate)**

- Warfarin, DOACs, heparin, etc.
- **Yes:** Coagulation Studies Necessary
- **No:** Go to Step 4

**5. Imminent Procedure Requires Coagulation Tests?**

- Lumbar puncture, major surgery, etc. (Consider specific procedures)
- **Yes:** Coagulation Studies Necessary
- **No:** Go to step 5

**6. Provisional diagnosis require coagulation studies?**

**Yes:** coagulation studies needed**.**

**No:** Go to Step 7

**7. Based on your review of the entier case, do you think coagulation studies are needed?**

**Yes:** coagulation studies needed**.**

**No:** coagulation studies are unncessary.

The END
